# Uniformities in Nature

**Published:** 1871-08

**Authors:** 


### UNIFORMITIES IN NATURE.

For six thousand years the sun has risen every morning at the appointed time, not varying the fraction of a second.

In some species of Indian corn, every ear has the same number of rows.

Every adult healthy man has the same number of teeth.

The common snail has always a hundred and eleven teeth in each row, and one hundred and ten rows, making twelve thousand two hundred and ten teeth in all; and thus it is in the entire range of created things: like begets like. Order is Heaven's first law; were it otherwise, chaos would reign throughout the universe. And a blessed thing it is that the omnipotent, the omniscient, and the omnipresent Creator of us all makes all things the special object of his peculiar care, from the whirling of the planets to the fall of a sparrow; and better still, that his loving kindness is over all his works.

